# Supplementary figures and images for: Simplified hypertension screening methods across 60 countries: An observational study
Source: PLoS Med. 2022 Apr 1;19(4):e1003975. doi: 10.1371/journal.pmed.1003975 (PMC9012386; doi:10.1371/journal.pmed.1003975)

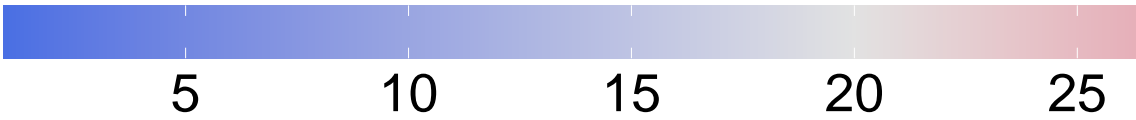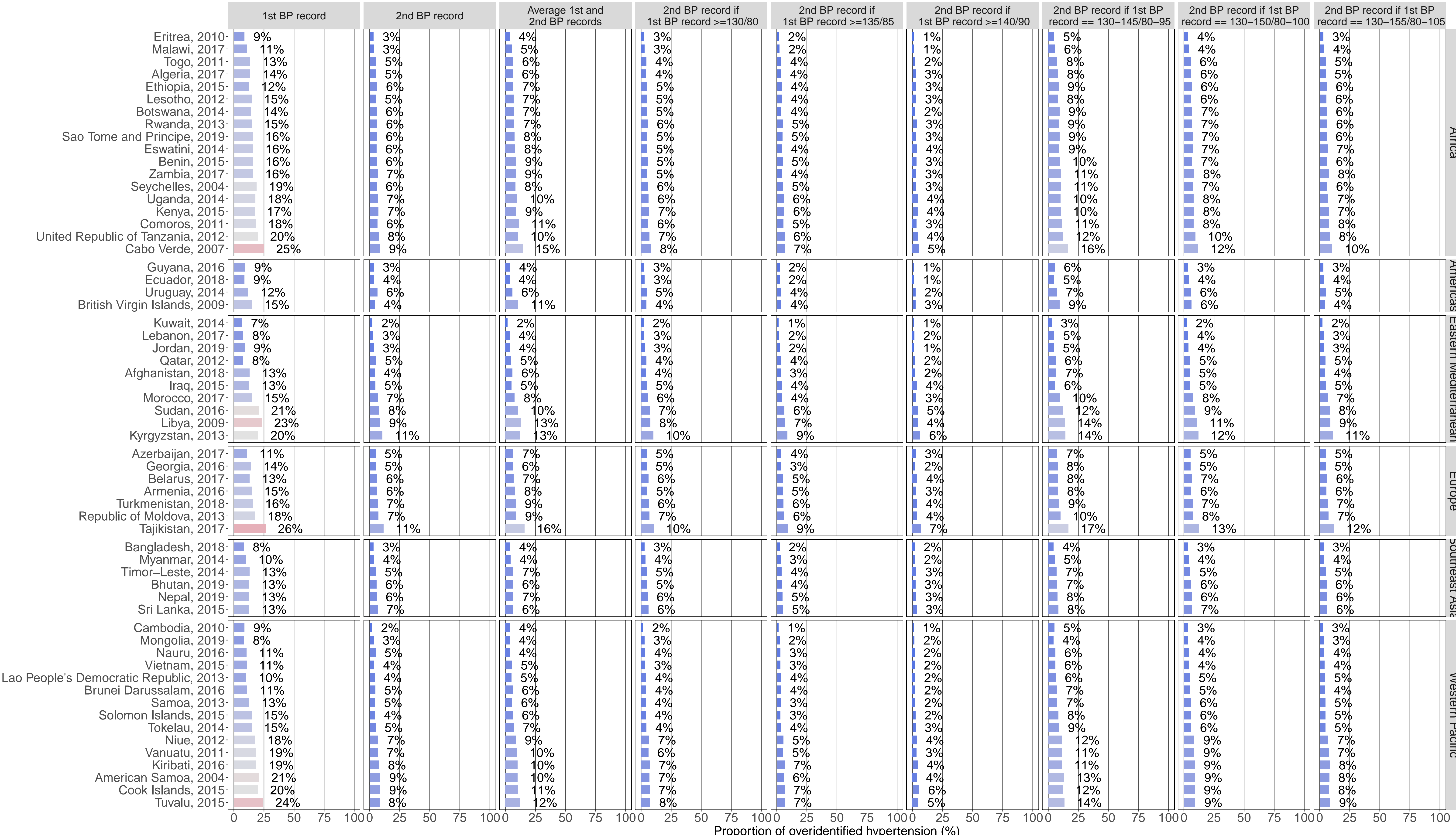

Proportion of overidentified hypertension (%)

Supplement: S1 Fig — (PDF) [file pmed.1003975.s004.pdf]

Significant difference with consistent HTN? ■ No ■ Yes

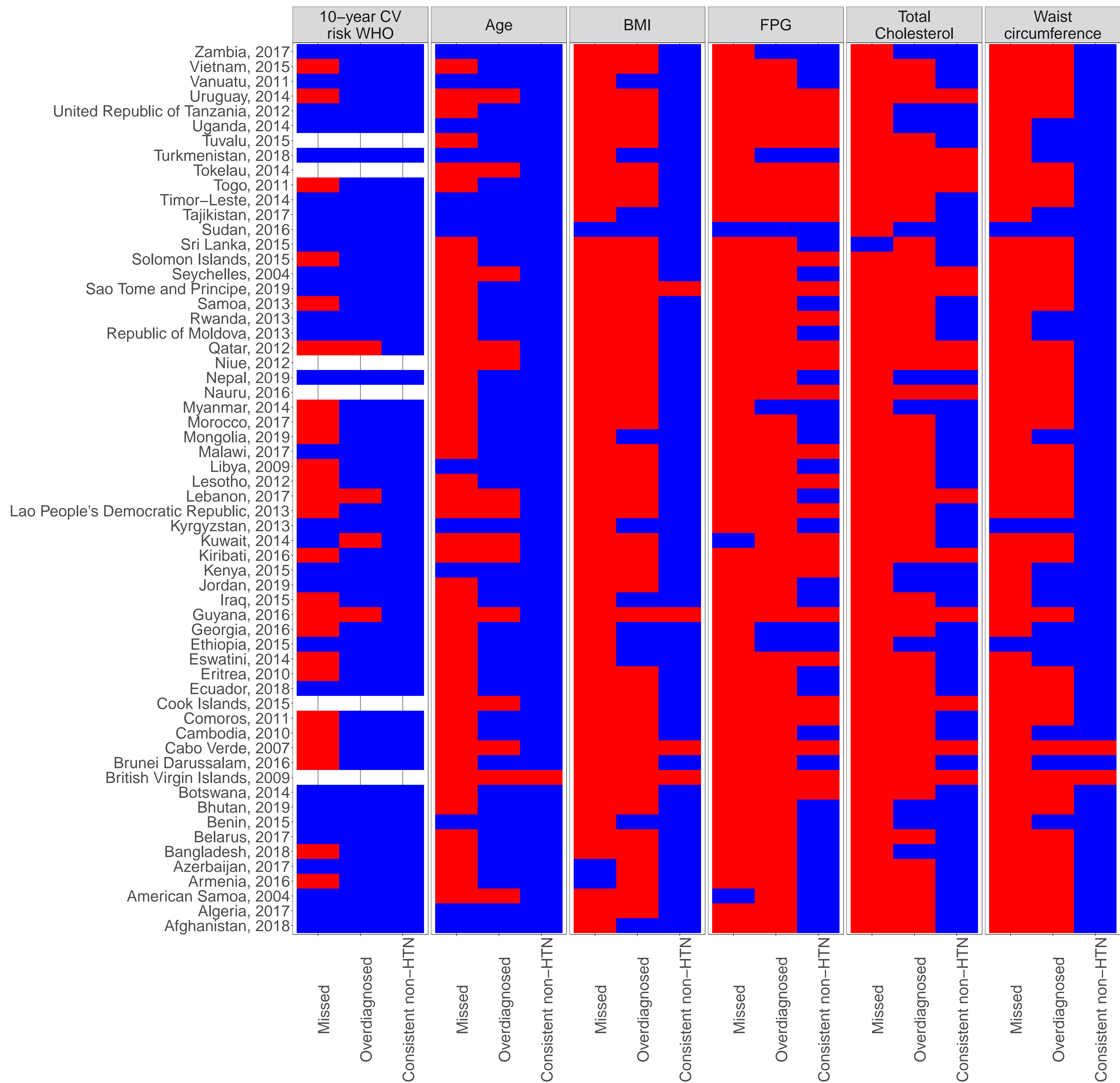

Supplement: S2 Fig — (PDF) [file pmed.1003975.s005.pdf]

Significant difference with consistent HTN? ■ No ■ Yes

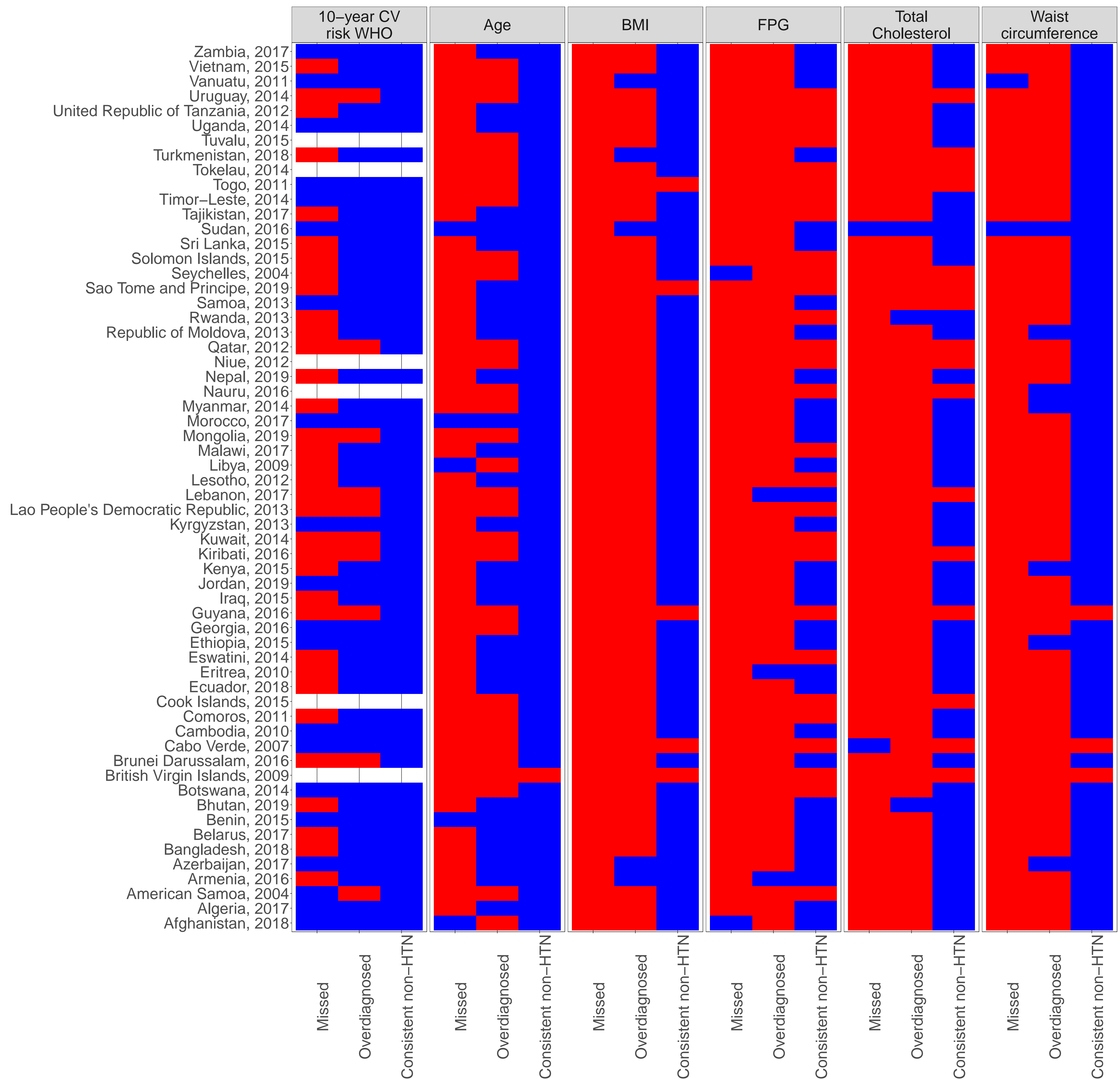

Supplement: S3 Fig — (PDF) [file pmed.1003975.s006.pdf]

Significant difference with consistent HTN? ■ No ■ Yes

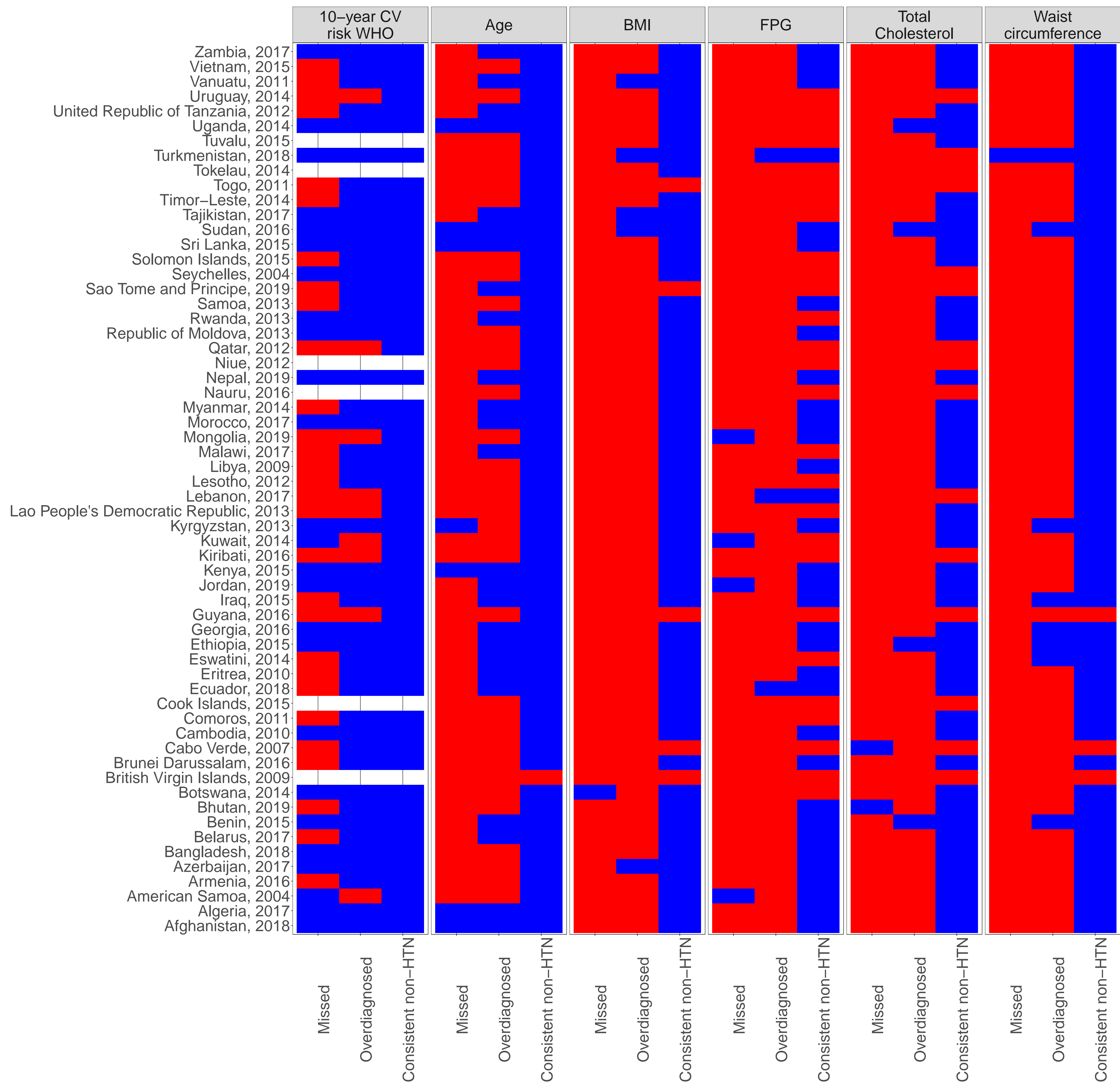

Supplement: S4 Fig — (PDF) [file pmed.1003975.s007.pdf]

Significant difference with consistent HTN? ■ No ■ Yes

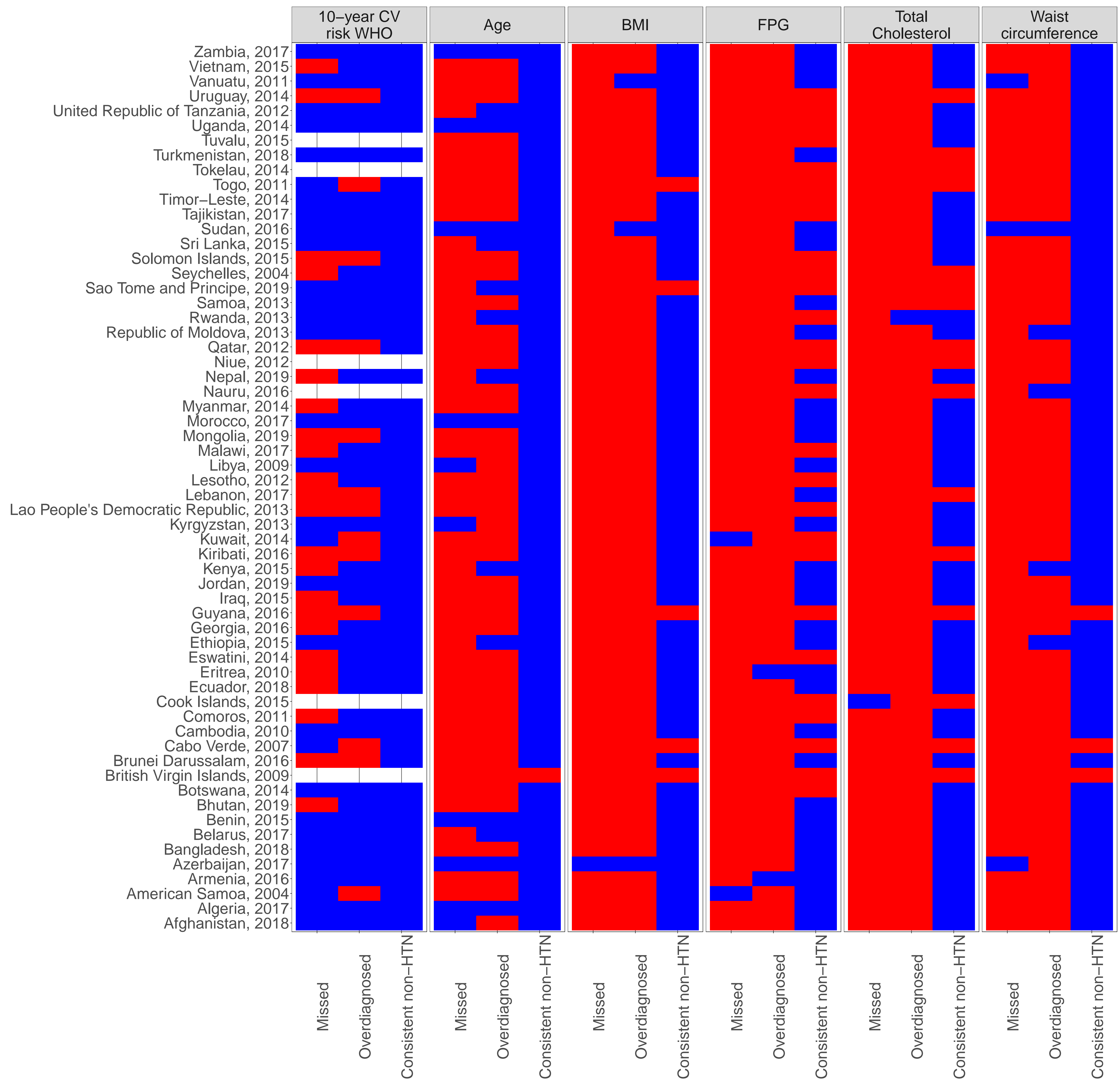

Supplement: S6 Fig — (PDF) [file pmed.1003975.s009.pdf]

Significant difference with consistent HTN? ■ No ■ Yes

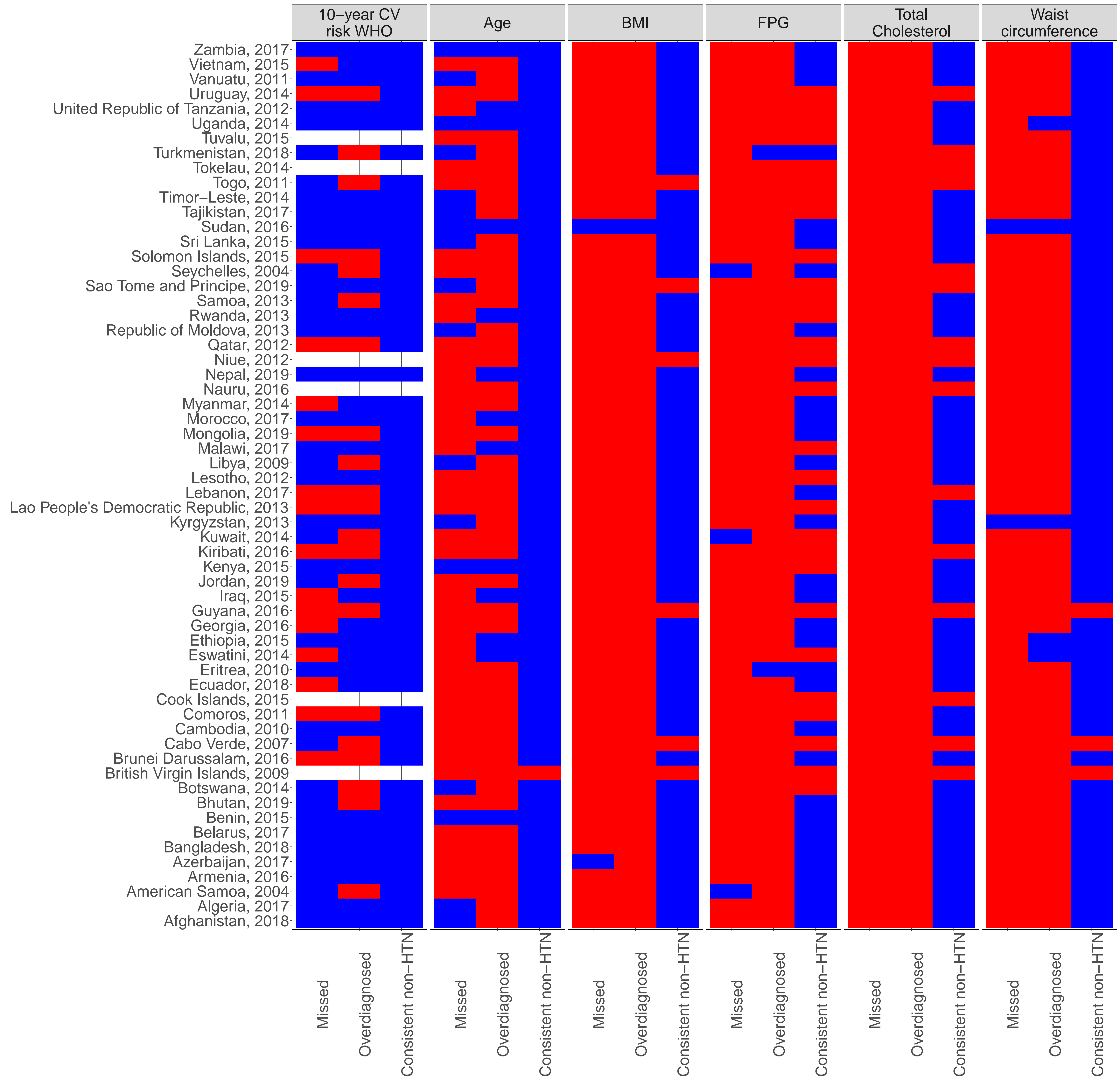

Supplement: S7 Fig — (PDF) [file pmed.1003975.s010.pdf]

Significant difference with consistent HTN? ■ No ■ Yes

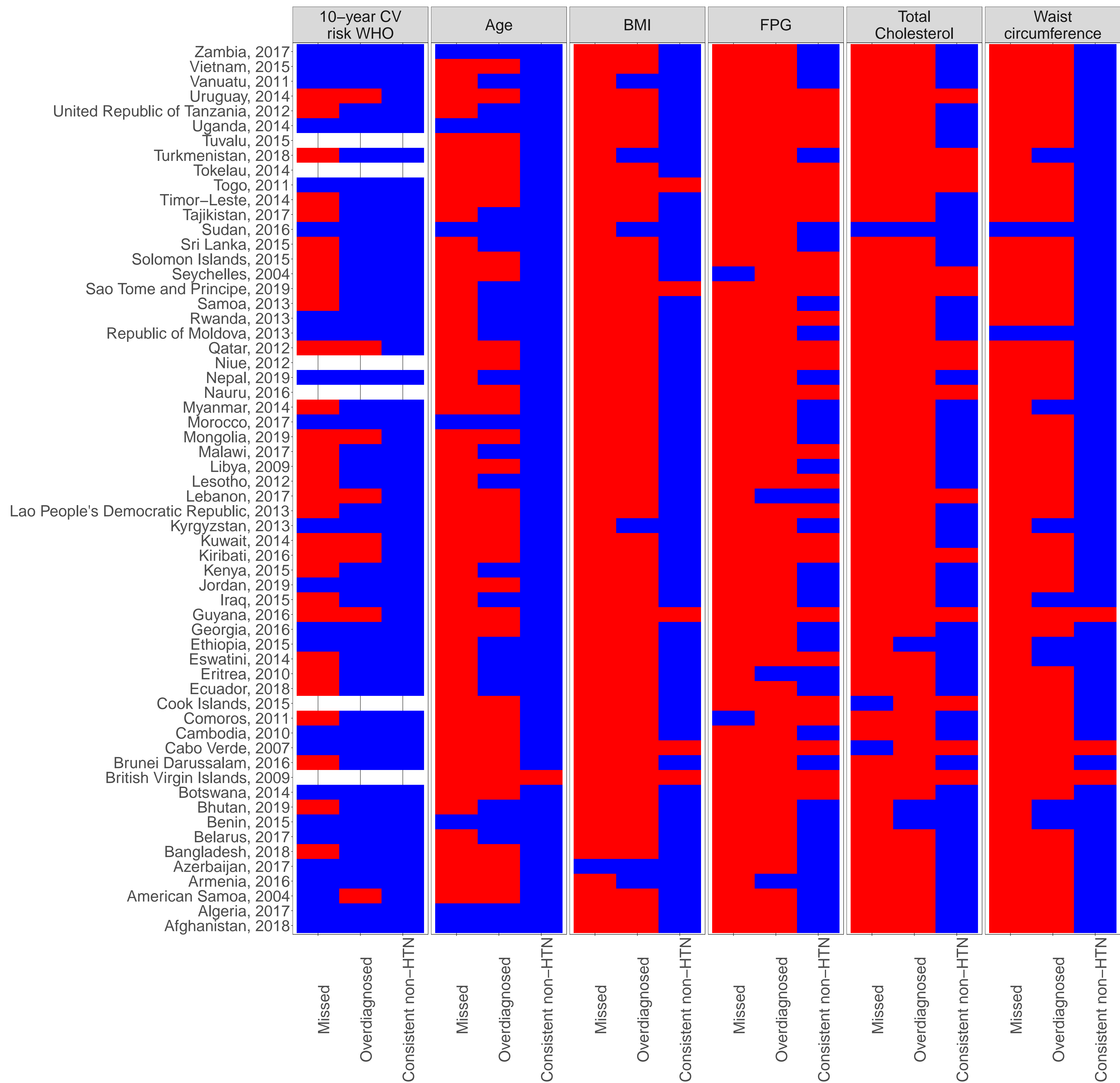

Supplement: S9 Fig — (PDF) [file pmed.1003975.s012.pdf]

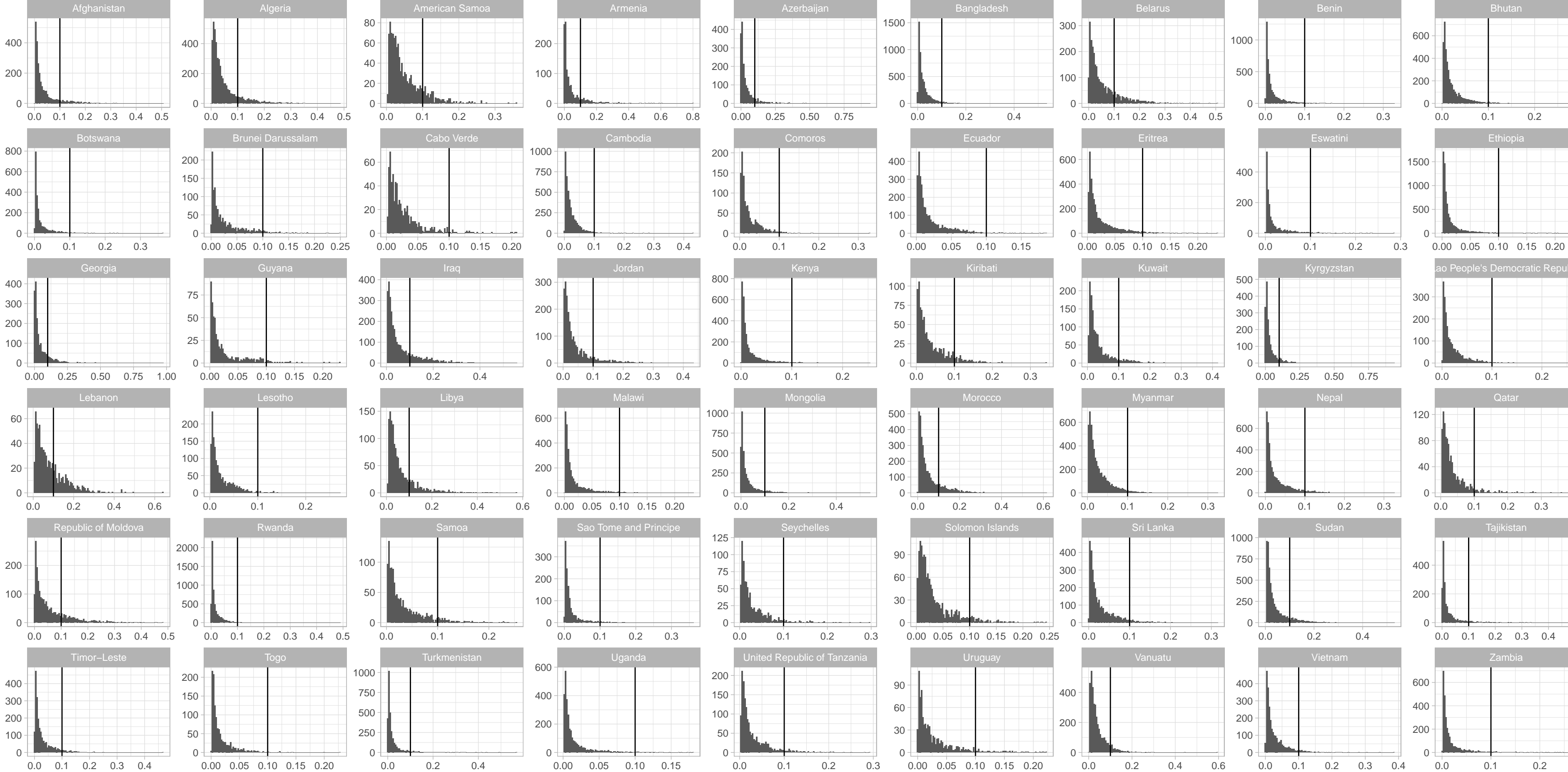

10-year cardiovascular risk

Supplement: S11 Fig — (PDF) [file pmed.1003975.s014.pdf]
