# Supplementary material for: Simplified hypertension screening methods across 60 countries: An observational study
Source: PLoS Med. 2022 Apr 1;19(4):e1003975. doi: 10.1371/journal.pmed.1003975 (PMC9012386; doi:10.1371/journal.pmed.1003975)
Supplement: S1 Supplementary Flow Chart — (DOCX) [file pmed.1003975.s015.docx]

278,586 people

75 countries

274,331 people

75 countries

18-69 years

251,793 people

74 countries

Missing any blood pressure data

Implausible and missing in blood pressure, body mass index, waist circumference, fating plasma glucose and total cholesterol

175,842 people

63 countries

175,796 people

63 countries

Pregnant women

145,518 people

63 countries

Self-reported and medication for hypertension

145,463 people

63 countries

Missing in smoking data

145,174 people

60 countries

Surveys with <200 observations

Preliminary data exploratory analyses showed that surveys with <200 observations yielded few observations in some groups (e.g., missed hypertension cases) and the p-values (t-tests) were not possible to calculate. We therefore decided to drop those surveys with <200 observations.
