# Supplementary material for: Simplified hypertension screening methods across 60 countries: An observational study
Source: PLoS Med. 2022 Apr 1;19(4):e1003975. doi: 10.1371/journal.pmed.1003975 (PMC9012386; doi:10.1371/journal.pmed.1003975)
Supplement: S5 Fig — (PDF) [file pmed.1003975.s008.pdf]

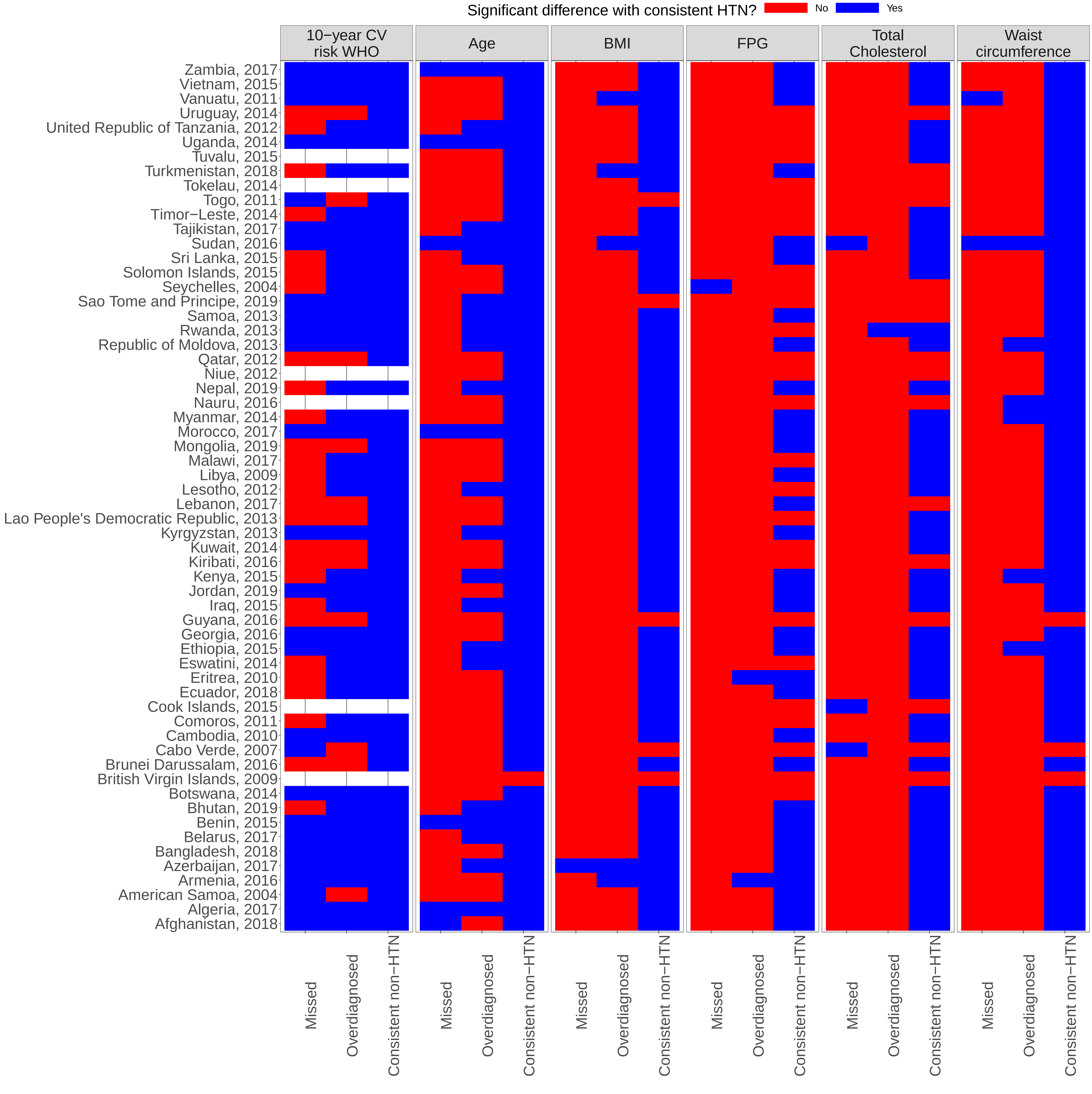

Significant difference with consistent HTN?
No
Yes
10−year CV risk WHO
Age
BMI
FPG
Total Cholesterol
Waist circumference
Zambia, 2017
Vietnam, 2015
Vanuatu, 2011
Uruguay, 2014
United Republic of Tanzania, 2012
Uganda, 2014
Tuvalu, 2015
Turkmenistan, 2018
Tokelau, 2014
Togo, 2011
Timor−Leste, 2014
Tajikistan, 2017
Sudan, 2016
Sri Lanka, 2015
Solomon Islands, 2015
Seychelles, 2004
Sao Tome and Principe, 2019
Samoa, 2013
Rwanda, 2013
Republic of Moldova, 2013
Qatar, 2012
Niue, 2012
Nepal, 2019
Nauru, 2016
Myanmar, 2014
Morocco, 2017
Mongolia, 2019
Malawi, 2017
Libya, 2009
Lesotho, 2012
Lebanon, 2017
Lao People's Democratic Republic, 2013
Kyrgyzstan, 2013
Kuwait, 2014
Kiribati, 2016
Kenya, 2015
Jordan, 2019
Iraq, 2015
Guyana, 2016
Georgia, 2016
Ethiopia, 2015
Eswatini, 2014
Eritrea, 2010
Ecuador, 2018
Cook Islands, 2015
Comoros, 2011
Cambodia, 2010
Cabo Verde, 2007
Brunei Darussalam, 2016
British Virgin Islands, 2009
Botswana, 2014
Bhutan, 2019
Benin, 2015
Belarus, 2017
Bangladesh, 2018
Azerbaijan, 2017
Armenia, 2016
American Samoa, 2004
Algeria, 2017
Afghanistan, 2018
Missed
Overdiagnosed
Consistent non−HTN
