# Supplementary material for: Simplified hypertension screening methods across 60 countries: An observational study
Source: PLoS Med. 2022 Apr 1;19(4):e1003975. doi: 10.1371/journal.pmed.1003975 (PMC9012386; doi:10.1371/journal.pmed.1003975)
Supplement: Attachment — Submitted filename: Rebuttal 20220301.docx [file pmed.1003975.s033.docx]

**NB: all page numbers refer to the version with track changes.**

**EDITOR**

**Q1. Please include line numbers in your next draft.**

R1. Included.

**Q2. Please revise your title according to PLOS Medicine's style. Your title must be nondeclarative and not a question. It should begin with main concept if possible and include the setting of the study. Please place the study design (e.g., “a multi-country evaluation of surveys”) in the subtitle (i.e., after a colon).**

R2. The title is not declarative and is not a question. The title begins with the main concept and include the setting of the study (p. 01): “*Simplified hypertension screening: a multi-country evaluation of surveys*”.

**Q3. Please structure your abstract using the PLOS Medicine headings (Background, Methods and Findings, Conclusions). Please merge Methods and Results into one section “Methods and Findings”.**

R3. Changed as requested.

**Q4. Please ensure that all numbers presented in the abstract are present and identical to numbers presented in the main manuscript text.**

R4. Verified.

**Q5. Please include the study design, regions and setting, total number of participants, month/year during which the surveys took place.**

R5. We have included the study design and the total number of participants. Because of the word limit for the abstract (300 words), it is impossible to give regions and years for each survey (because there are 60 surveys). However, we included the following line with a global summary: “*We used 60 WHO STEPS surveys (60 countries, cross-sectional and nationally representative; n=145,174) from six world regions conducted between 2004-2019.*”

**Q6. Please include the specific important dependent variables that are adjusted for in the analyses.**

R6. Included: “*… (adjusted for age, sex, body mass index and 10-year cardiovascular risk) …*”

**Q7. In the last sentence of the Abstract’s Methods and Findings section, please describe the main limitation(s) of the study's methodology.**

R7. Included: “*The main limitation of this work is the cross-sectional design.*”

**Q8. Abstract Conclusions: Please address the study implications without overreaching what can be concluded from the data.**

R8. Modified as suggested; one new sentence was included at the end: “*Simplified BP screening approaches could be included in large screening programs and busy clinics.*”

**Q9. At this stage, we ask that you include a short, non-technical Author Summary of your research to make findings accessible to a wide audience that includes both scientists and non-scientists. The Author Summary should immediately follow the Abstract in your revised manuscript. This text is subject to editorial change and should be distinct from the scientific abstract. Please see our author guidelines for more information:** [**https://journals.plos.org/plosmedicine/s/revising-your-manuscript#loc-author-summary**](https://journals.plos.org/plosmedicine/s/revising-your-manuscript#loc-author-summary)

R9. Included, right after the abstract and before the Introduction.

**Q10. Introduction: Please conclude the Introduction with a clear description of the study question or hypothesis.**

R10. Included: “*We aimed to answer these research questions, what are the misdiagnosis and over-diagnosis rates according to nine simplified blood pressure screening approaches? And what is the underlying cardiovascular risk profile in these groups (e.g., misdiagnosed, over-diagnosed and consistently diagnosed)?*”

**Q11. Did your study have a prospective protocol or analysis plan? Please state this (either way) early in the Methods section.**

R11. This has been acknowledged early in the Method section (p. 05). “*None of the analyses presented in this paper were prespecified in a protocol.*”

**Q12. Please ensure that the study is reported according to the STROBE guideline and include the completed STROBE checklist as Supporting Information. Please add the following statement, or similar, to the Methods: "This study is reported as per the Strengthening the Reporting of Observational Studies in Epidemiology (STROBE) guideline (S1 Checklist)." The STROBE guideline can be found here:** [**http://www.equator-network.org/reporting-guidelines/strobe/**](http://www.equator-network.org/reporting-guidelines/strobe/)

R12. The suggested text was included (p. 05): “*This study is reported as per the Strengthening the Reporting of Observational Studies in Epidemiology (STROBE) guideline (Supplementary Checklist).*” The STROBE checklist for cross-sectional studies is being uploaded as a new Supplementary Material

**Q13. Please provide 95% CIs and p values, where relevant, for all estimates in the text and tables.**

R13. The 95% confidence intervals and p-values are reported in tables and/or supplementary tables.

**Q14. Please do not report p values to 3 decimal points**

R14. Amended.

**Q15. In the Discussion section, please delete the titles “results in context” and “clinical medicine and public health implications”**

R15. Deleted.

**Q16. Please define the abbreviations in Tables and Figure e.g., BMI in Table 5.**

R16. Defined.

**Q17. Please remove the “Conflict of Interests”, “Data availability,” and “Funding” statements at the end of the main text. This information is captured in the metadata obtained in the submission form.**

R17. Removed.

**Q18. References.**

R18. Modified as suggested.

**REVIEWER #1**

**This is a well-conducted study on simplified hypertension screening with regard to misdiagnosis, over-diagnosis and cardio-metabolic characterization in 60 low- and middle-income countries. The study design, datasets, and statistical methods and analyses are mostly adequate. However, there are still a few issues needing attention, especially in presentation of the results.**

We appreciate the positive feedback by the reviewer and are grateful for her/his time invested in reviewing our work. We have done our best to incorporate her/his suggestions.

**Q1. The study is mostly descriptive and would be good to keep it descriptive to present all the facts on missing hypertentions in the 9 simplified screening approaches. The 10-year cardiovascular risk is just a risk score which itself has limitations in validation and accuracy, and they are a few others of similar kind. As all the surveys are cross-sectional studies, it would be good to focus on the observed facts rather than projections. Both Table 4 and Figure 2 are huge and not very informative and also on 10-year cardiovascular risk, suggest to move both to the supplementary information, and also tone down the claims on 10-year risks as subject to scrutiny and more work is needed.**

R1. The 10-year cardiovascular predicted risk was not meant to inform about the future (i.e., projections). We used it as a “summary” of cardiovascular risk. This way, the results focused on the 10-year cardiovascular risk rather than on body mass index, total cholesterol, diabetes prevalence and smoking at a time. Had the narrative of our work focused/described each cardiometabolic risk factor independently (rather than the 10-year predicted as an overall measure of cardiometabolic risk), the manuscript would have been much longer. To avoid over-emphasizing the role of the 10-year cardiovascular risk, we included the following lines in the Methods (p. 06):

“*We did not intend to make projections about the cardiovascular risk; rather, we used the 10-year cardiovascular risk as a summary measure to characterize the overall cardiometabolic profile according to the groups of interest. Alternatively, we would have had to describe each cardiometabolic risk factor, making the results cumbersome.*”

Furthermore, we also discussed about this matter in the Limitations subheading (p. 15):

“*We used a standard cardiovascular risk score recommended for global use; however, this score may still have limitations. We used the cardiovascular risk score as a summary measure to characterize the overall cardiometabolic risk profile, not to make predictions about the cardiovascular outcomes in these populations. As discussed above, longitudinal studies are needed to characterize the long-term cardiovascular outcomes according to the simplified blood pressure measurement approaches.*”

As suggested by the reviewer, Table 4 and Figure 2 were moved to the Supplementary Materials. The results were updated accordingly.

**Q2. Only around a couple of thousands participants in the survey in each country which is subject to variation and bias for the estimates. Can authors please go a bit comprehensive and critical in the limitation section to discuss the potential impact on the results of the study?**

R2. We elaborated about this matter in the Limitations subheading (pp. 15-16):

“*Fourth, even though we pooled 60 national health surveys, all of which were nationally representative, for some countries the analysis included approximately 2,000 individuals after we applied our selection criteria. A sample size of ~2,000 people could be considered (rather) small, which could lead to variations in the estimates. However, we argue that these surveys were conducted using standard and validated complex survey design in a random sample of the general population. Therefore, they provide informative results for the overall population in these countries. Given our selection criteria, the results for each country may not be representative of all people in the target population, but our results strongly characterize the patterns and profiles of the nine simplified blood pressure measurement strategies.*”

**Q3. Table 5 on factors contributing to missed hypertension diagnosis. Age and BMI are mostly consistent but cadiovascular risk is not really, only 4 of 9 have significant results. Any interpretations and cautions to read the results?**

R3. We elaborated about this subject in the Discussion section (pp. 11-12):

“*The regression coefficients for the absolute cardiovascular risk in some of the models, depending on the simplified approach, did not reach statistical significance. We would argue that this signalled groups in which the cardiovascular risk was not (truly) associated with misdiagnosis. Given the large sample size included in the models, the results most likely show strong associations (or lack of association) than unstable results. Nevertheless, residual confounding could still be a possibility, even though we included relevant and potential confounders available. Our results must be further validated with larger samples and, most importantly, with prospective cohort studies to examine the mid/long-term cardiovascular outcomes of the missed and consistent hypertension groups.*”

**REVIEWER #2**

**This is a very well written, clear, and important paper. The authors identified the share of misdiagnoses and over-diagnoses when using simplified hypertension screening approaches. Given that hypertension screening needs to be increased globally in the fight against the epidemiological transition, more information on how screening efforts can be increased and made more efficient are highly relevant. The authors use adequate statistical methods in answering their research question. I have only minor comments and suggestions - and the authors may choose to ignore them.**

We appreciate the reviewer enjoyed our work. We are happy to have incorporated her/his suggestions.

**Q1. One sentence is a bit unclear (page 4 and 5): "We used the same predictors except for diabetes; the original model included history of diabetes whereas we included history and new diabetes cases (i.e., aware, and unaware cases, the latter defined with fasting plasma glucose ≥126 mg/dL)" It should be made clearer which predictors this is referring to.**

R1. We meant that we used the 2019 WHO Cardiovascular Risk Charts with the same predictors as they included in the original derivation of the model, except for diabetes. In other words, for our calculations we used the same predictors as in the original 2019 WHO Cardiovascular Risk Charts except for diabetes. Regarding the diabetes, the original model only included history of diabetes, whereas we included history and new diabetes cases (as defined by fasting plasma glucose ≥126 mg/dl). We rewrote these sentences to make the message clearer (p. 05):

“*To compute the 10-year predicted absolute cardiovascular risk, we used the same predictors as in the original 2019 WHO Cardiovascular Risk Charts except for diabetes; the original model included history of diabetes whereas we included history and new diabetes cases (i.e., aware, and unaware cases, the latter defined with fasting plasma glucose ≥126 mg/dl). We made this decision to characterize the highest cardiovascular risk profile in the missed and over-diagnosed populations in low- and middle-income countries (LMICs), where many people with diabetes are still unaware of their diagnosis.*”

**Q2. Perhaps the authors could add more details on how consequential simplified hypertension screenings may actually be for the CVD disease burden. This could be done by:**

**- including references for HTN screening being still too low in LMICs (see this study** [**https://www.thelancet.com/pdfs/journals/lancet/PIIS0140-6736(19)30955-9.pdf**](https://www.thelancet.com/pdfs/journals/lancet/PIIS0140-6736(19)30955-9.pdf) 
            **- discussing how relevant it actually is to make screening time shorter. I wonder how much time is being saved by taking only e.g., only one measurement (could briefly mention wait time in between measurements). As the authors have pointed out, this may be particularly relevant in massive screening programs - but how frequent are these or should even be as screening efforts are being ramped up?**

R2. The suggested reference has been included in the Introduction section (p. 04): “*…where screening for hypertension remains suboptimal.*” Also, we included this reference in a new clause in the Discussion section (p. 12): “*…particularly in LMICs where screening for hypertension is still limited.*”

We also discussed this subject (p. 11): “*The WHO STEPS protocol, as other similar guidelines, recommend waiting three minutes between blood pressure measurements. If there are three measurements (standard), and we assume that each measurement takes seconds, then measuring blood pressure in one subject could last for at least six minutes. This would be equivalently to measuring blood pressure in 10 people per hour. However, if a simplified approach is implemented, whereby for example only two measurements are required, the time invested in one blood pressure measurement would be three minutes approximately. In other words, we could measure blood pressure in 20 people per hour, substantially increasing the number of individuals who could be screened for hypertension. The simplified approaches could sae 50% of time needed to measure blood pressure in one person compared to current and standard guidelines.*”

There is a global hypertension screening initiative which runs every year since 2017: May Measurement Month (MMM; <https://maymeasure.com/>). To date, this program has covered >100 countries and benefited >1,000,000 people. Arguably, and pending further verification, the simplified approaches we studied could be implemented in future MMM so that they can (even) reach more people.

**REVIEWER #3**

**This is an interesting approach to an important question. It's a big paper with a lot of analyses. I have one substantial criticism and a few smaller ones.**

We appreciate the reviewer found merit in our work. We did our best to incorporate her/his comments.

**Q1. The substantial criticism, which the authors responsibly describe in their limitations, is that the protocol-driven hypertension assessment of STEPS has poor alignment with real-world BP practices. A fair amount of evidence shows clinic values are less reliable, wider variability, and generally don't do the three-measure assessment this study is examining. It's testing a measurement approach that I basically think doesn't exist in the world. This makes the external utility of this work unclear to me.**

R1. Certainly. The three-measurement protocol may not be strictly followed everywhere under “real-world” circumstances. Notwithstanding, there are places and circumstances where the three-measurement protocol is followed.^[1],^^[2],^^[3]^ Our work could have real utility in such circumstances, and we would like to rise these three potential applications.

1. May Measurement Month (MMM; <https://maymeasure.com/>). Since 2017, and on a yearly basis, this global (and massive) screening program has reached >100 countries and >1,000,000 people (only in 2016-17, 1,201,570 people in 80 countries participated in MMM^[4]^). This global screening program follows the three-measurements protocol. Our work could inform future MMM (and alike initiatives), so that they take fewer measurements, saving time and consequently reaching more people. This is, we believe, a strong and palpable utility of our work.
2. Research, large population-based or national health surveys. The STEPS Surveys have been applied for multiple years in several countries following the three-measurement protocol. Other independent national health surveys, as well as other global surveys (e.g., Demographic and Health Surveys), have adopted and/or adapted the WHO Steps protocol and may also take three blood pressure measurements. Our work could inform these surveys and spark the discussion on whether three measurements are needed, or an alternative approach could be implemented to save time and resources.
3. Busy clinics. Some clinics and other healthcare facilities may have few personnel, the personnel may need to do multiple tasks (thus unavailable for blood pressure measurements), or there may be few sphygmomanometers. These constraints, often seen in low/middle-income countries, could benefit of simplified approaches to measure blood pressure. Our work could provide these settings with pragmatic alternatives to measure blood pressure reaching more people than under normal circumstances. In other words, the simplified approaches could make it easier to reach more patients, despite the shortage of personnel or lack of medical devices.

These arguments about the potential applications of our work have been elaborated in the Discussion section of our revised manuscript (p. 12):

“*Therefore, the potential applications of our work target two relevant scenarios. First, May Measurement Month. This is a global hypertension screening program yearly conducted, and since 2016 it has covered >100 countries benefiting >1,000,000 people. In this program they follow the three-measurement protocol. Our work could inform future May Measurement Months motivating the discussion on whether fewer blood pressure measurements can be taken to maximize resources while reaching a much larger population. Second, our work could also influence future research and large health surveys. In addition to the WHO STEPS Surveys themselves, the WHO STEPS Survey protocol has influenced other population-based health surveys worldwide which would also take three blood pressure measurements. Our work could spark interest in discussing whether three BP measurements are needed, or fewer measurements are a reasonable option to save resources which could be used to measure other relevant health variables. Third, in some clinics there may be lack of sphygmomanometers or shortage of personnel, limiting the number of people who can be screened for hypertension. Our work could deliver pragmatic approaches to optimize the protocols for BP measurement, to maximise the number of people who can be screened.*”

**Q2. The smaller complaints all stem from, to my reading, them not quite succeeding at making a hard topic simple enough for the reader. There are a lot of analyses and I never quite found the "story" in their outcomes. There are 3 things that make this hard to write about. First is that diagnostic testing is a difficult field. The established terms of sensitivity and specificity are confusing and unnatural. Seemingly to avoid that they used non-standard terms like proportion missed and over-diagnosed. But these were hard to track and weren't reported consistently. The core findings - the classic 2x2 table of missed and overdiagnosed relative to true and non-hypertensive - was clear in the abstract, but not elsewhere.**

R2. We appreciate the reviewer shared with us her/his writing style. We have completely written the Results section. Please, kindly refer to the revised manuscript to read the new Results section. A summary of what the reader will find in each subheading of the Results section is provided below.

1. First subheading (Data description). We summarised relevant statistics to become familiar with the data we analysed.
2. Second subheading (Misdiagnosis). We changed the story around the two simplified approaches that had the smallest misdiagnosis rates. We developed the narrative from general (worldwide) to specific (country). We closed this subheading with a “take-home” message, sort of a summary of which approach appear to be better and why.
3. Third subheading (Over-diagnosis). We changed the story around the simplified approaches that had the smallest over-diagnosis rates. We elaborated the narrative from general (worldwide) to specific (country). We closed this subheading with a “take-home” message, in which we combined these findings with those from the previous subheading (misdiagnosis) and gave a “take-home” message about the most relevant simplified approaches.
4. Fourth subheading (Cardiovascular risk profile amongst the misdiagnosed cases). We elaborated, also around the best-performing simplified approaches, whether the mean 10-year cardiovascular risk would be higher/lower amongst the missed cases. We closed this subheading with a “take-home” message about whether the missed cases have or not higher 10-year cardiovascular risk.
5. Fifth subheading (Cardiovascular risk profile amongst the over-diagnosed cases). We elaborated, around the best-performing approaches, whether the mean 10-year cardiovascular risk would be higher/lower amongst the over-diagnosed cases. We finished this subheading with a “take-home” message about whether the over-diagnosed cases have or not higher cardiovascular risk.
6. Sixth subheading (Potential correlates for missed hypertension cases). We described the regression models while commenting on the pragmatic implications of these findings.

We have avoided using terms like “sensitivity” and “specificity” and are consistently reporting “misdiagnosis” and “over-diagnosis” throughout the manuscript. As it is the case with other similar literature,^[5]^ we believe that using “misdiagnosis” and “over-diagnosis” could be much more friendly for a broader audience. These terms are almost self-explanatory, referring to people that should have been diagnosed but were not with the new approach (misdiagnosed), and referring to people that should have not been diagnosed but were with the new approach (over-diagnosis). These friendlier definitions have been included in the Methods section (p. 06):

“*In simple terms, misdiagnosis would refer to people who should have been diagnosed with the standard approach but were not with the new approach; similarly, over-diagnosis would refer to people who should have not been diagnosed with the standard approach but were with the new approach.*”

**Q3. Second, cross-national comparisons are hard to write because it's hard to see the story. A table of 60 countries is very hard to get a feel for or read, especially because it's unlikely that 2 countries of the same region actually have different phenomena. The manuscript dealt with this difficulty 3 different ways. They had a few tables with 60 countries, a few divided by continent, and some organized internationally with multilevel modeling (Table 5). I can see the urge for all of these, but it didn't help me understand what was going on. What do we learn from each? Do we think it's a national, regional, or single international phenomenon?**

R3. The original “story” in the manuscript was around the countries and we described the lowest, highest and means across countries. The new “story” in our results refer to the best-performing simplified approaches. Countries are occasionally named to give some examples or to support some argument. Please, refer to our previous response and to the new Results section in the revised submission.

**A4. This became especially difficult in the figures, which don't really work for me. They showed statistical significance, which is a feature of sample size, effect size, and random chance. In this case we know the effect is real - that the new measures do not precisely match the old - so there's no question we'd reach significance with enough sample. But I don't know the sample size of each of the 60 rows and this obscures the effect size, which is more interesting. I generally didn't find the story behind the figures that compelling compared to the general issue of learning how reliable the techniques are. (Though the fact, which appeared in a few ways, that the errors were larger in those with high risk and history of CVD is both concerning and interesting.)**

R4. As suggested by another reviewer, some of the tables and figures that were originally in the manuscript have been moved to the Supplementary Materials. Also, the Results now elaborate, narrate, and explain the results rather than summarizing (or repeating what was shown in the figures). About the sample size, which provide relevant information to better understand the differences, Supplementary Tables 5 to 13 show the sample size per group (i.e., misdiagnosed, or over-diagnosed) and by country, together with the exact p-values of the t-test comparisons.

**Q4. The third difficulty was just that nine is a lot of comparisons. This made some tables feel overwhelming. But to address that, they didn't include all 9 at all spots in the paper, like the figures, even though we'd just seen them in a table.**

R4. Certainly, nine simplified approaches may seem overwhelming. We have rewritten the Results section focusing on two of the simplified approaches, while the rest are available for the interested reader (Supplementary Materials). We trust the reviewer will find the new Results section closer to the style she/he suggested.

**Q5. I think if this paper is resubmitted, I would recommend really deciding what the story is and telling that. Then, putting the details in the supplement and acknowledging in the paper that what is presented is post-hoc. I would put the missed and overdiagnosis in the same tables. I would report sensitivity and specificity somewhere. I would recommend grouping the countries in a way that feels meaningful and only putting all 60 in the main paper once. (Put more in an appendix.)**

Q5. We did our best to incorporate the reviewer’s style into the paper. We believe her/his recommendations have substantially improved our work. We appreciate the time she/he invested in reading and commenting our work.

**Q6. Table 5 seems to show that higher risk people are likely to have missed HTN. But Table 4 seems to show that missed hypertension have a risk of CVD that is roughly half the risk of those with consistent hypertension. How?**

R6. Table 5 shows that in five models (i.e., in five out of nine simplified approaches), there is a significant positive association with 10-year cardiovascular risk. For example, the model for the simplified approach based on the second BP measurement only was not significant, and in Table 4 there were 13/60 countries in which the cardiovascular risk is <50% the risk of those in the consistent hypertension; the smallest proportion being 22%. With 13 countries and a ratio of 22%, it may seem expected to have a nonsignificant association with the 10-year cardiovascular risk in the regression models. On the other hand, the model for the simplified approach based on the first BP measurement only was significant, and in Table 4 there were 13/60 countries in which the cardiovascular risk is <50% the risk of those in the consistent hypertension; the smallest proportion being 34%. With 13 countries also though a ratio of 34% (vs 22% above), it may be expected that the association was significant in the regression model.

**Q7. In table 5 the HR of CV risk is ~3 in all examples except one where it's 0.89. Is that right?**

R7. Yes, this is correct. The results have been verified.

**Q8. I would benefit by having them talk through the implications of the random effects findings in table 5.**

R8. This has been included in the new Results section (pp. 11-12):

“*The variability of the regression models was always larger between countries within regions, than between regions (consistent with Figure 1). This may imply that country-specific guidelines are needed to follow simplified approaches, rather than one guideline for all countries in a region.*”

This is also available in the Discussion section (p. 14):

“*Our results showed large variability across countries within world regions. While simplified BP screening approaches may be a sensible and pragmatic alternative to screen large populations, a one-size-fits-all simplified approach may not be possible. Countries may need to find the optimal trade-off between number of BP measurements and hypertension cases missed…*”

**Q9. Table 2 could use something like "by country" somewhere. I struggled to understand what the SD was the SD of, though I now see it's of the results of each country.**

R9. The following sentence has been included as a footnote to Table 2:

“*All estimates shown in the tables (mean, SD, median, minimum, and maximum) are across the 60 countries included in the analysis.*”

**Q10. I would probably merge tables 2 and 3, remove the min and max, and add columns for overdiagnosis into that table.**

R10. If the reviewer kindly allows, we will rather keep the two tables to avoid having one larger table.

**REVIEWER #4**

**Blood pressure measurement is key in the screening and management of high blood pressure. In the most comprehensive scenario patients would need multiple BP measurements on separate occasions, and in the most pragmatic approach would require three BP measurements and taking the average of the last two which is standard approach. Three BP measurement may however be challenging in resource-constraint settings because of shortage health care workforce. Therefore, it will be necessary to have fewer BP measurements that would have values as close as possible to taking the average of the last two measurements which is currently the gold standard. Although attempts have been made to find simplified BP screening approaches, these simplified approaches were tested in three countries only and BP may vary between countries. The authors then decided to analyse national surveys in 60 low- and middle-income countries giving a large dataset. The authors further went ahead to relate the different measurement to their effect on cardiovascular risk score. This paper is well written and a stimulation at looking for ways that can make blood pressure measurement especially during mass screening programmes to be more pragmatic.**

We feel enthusiastic about the positive comments by this reviewer. We appreciate the time invested in comprehensively reading our paper.

**Q1. Apart from country-to-country variations as stated by the authors is there any regional differences to the variation? Although one-size-fits will be applicable to different countries it will be nice as a more pragmatic approach for the authors based on their study to state the 2 or 3 methods out of the nine tested that they will recommend for countries to be tested.**

R1. As suggested, in the Discussion section we included a new paragraph in which we state two methods out of the nine tested that we will recommend for countries to be test. The new paragraph is on page 11 and reads:

“*The results highlight that some of the nine simplified approaches may lead to little misdiagnosis and are not associated with higher overall cardiovascular risk; however, we cautiously believe that further validation of these simplified approaches is warranted. Large prospective studies are needed to study the long-term cardiovascular outcomes for each simplified approach. Nevertheless, we would cautiously suggest considering two simplified approaches: i) only the second BP measurement; or ii) the second BP reading if the first BP measurement was between 130-145/80-95 mmHg. If deemed necessary by local experts, these two simplified approaches could be implemented in screening programs. Also, these two simplified approaches could be subject of further in-country validation analyses.*”

**REVIEWER #5**

**Q1. Authors considered only BP measurement for hypertension diagnosis but currently having BP lowering drugs, earlier suggestion by a practitioner about hypertension as well as lifestyle modification for hypertension management are part of hypertension definition which are ignored by authors.**

R1. Indeed, in most epidemiological studies about hypertension, this condition is defined as a composite variable including: i) blood pressure measurements; ii) self-reported diagnosis of hypertension; and ii) self-reported medication for hypertension (i.e., antihypertensives). In our study, we only considered blood pressure measurements. In fact, people who were taking antihypertensive medication or who were aware they had hypertension, were excluded from the analysis. This was reported in the Methods section, page 05: “*We included adults aged 18-69 years and free of known hypertension (self-reported medical history and antihypertensive medication).*”; this was also reported in the Flowchart in the Supplementary Materials.

We made this decision -to excluded people with known hypertension- because:

1. Simplified blood pressure measurements are to be used in (large) screening programs for hypertension (this argument was elaborated in the Introduction); for example, May Measurement Month (<https://maymeasure.com/>). Screening programs for hypertension target people without hypertension. Therefore, we limited the analysis to hypertension-free people, to simulate a real-life screening program to which hypertension-free people would go to have their blood pressure measured.
2. Had we included people taking antihypertensive medication, or people aware they have hypertension, could have biased the results for the general hypertension-free population attending hypertension screening programs. This, because the blood pressure of people with hypertension would be biased by their pharmacological and non-pharmacological treatment.

We introduced these two arguments in the Methods section (p. 05): “*In other words, we excluded people with hypertension because the simplified approaches for blood pressure measurements are meant to be used in large screening programs targeting the general population with undiagnosed hypertension.*”

Finally, this decision is also consistent with the latest global literature on simplified blood pressure measurement approaches.^[6]^

If we have aimed to report the prevalence of hypertension in these 60 countries, the exclusion of people with hypertension would be a limitation. However, our work aimed to quantify and characterize a set of simplified blood pressure measurement approaches amongst the hypertension-free general population.

**Q2. Reference 15 seems incorrect or broken.**

R2. All references have been verified and fixed as needed.

1. ACC/AHA/AAPA/ABC/ACPM/AGS/APhA/ASH/ASPC/NMA/PCNA Guideline for the Prevention, Detection, Evaluation, and Management of High Blood Pressure in Adults: A Report of the American College of Cardiology/American Heart Association Task Force on Clinical Practice Guidelines. J Am Coll Cardiol 2018 15;71(19):e127-e248.
2. 2018 ESC/ESH Guidelines for the management of arterial hypertension: The Task Force for the management of arterial hypertension of the European Society of Cardiology and the European Society of Hypertension: The Task Force for the management of arterial hypertension of the European Society of Cardiology and the European Society of Hypertension. J Hypertens 2018;36(10):1953-2041..
3. WHO Steps Surveillance Manual 2017. [cited 18 Oct 2021]. Available: https://www.who.int/ncds/surveillance/steps/STEPS_Manual.pdf
4. Beaney, T., Schutte, A. E., Tomaszewski, M., Ariti, C., Burrell, L. M., Castillo, R. R, et al. May Measurement Month 2017: an analysis of blood pressure screening results worldwide. The Lancet Global health 2018;6(7), e736–e743.
5. Tang O, Kou M, Lu Y, *et al.* Simplified hypertension screening approaches with low misclassification and high efficiency in the United States, Nepal, and India. *J Clin Hypertens Greenwich Conn* 2021.
6. Tang O, Kou M, Lu Y, *et al.* Simplified hypertension screening approaches with low misclassification and high efficiency in the United States, Nepal, and India. *J Clin Hypertens Greenwich Conn* 2021.
